# Patients with Primary and Secondary Bile Duct Stones Harbor Distinct Biliary Microbial Composition and Metabolic Potential

**DOI:** 10.3389/fcimb.2022.881489

**Published:** 2022-04-25

**Authors:** Ru Feng, Tianyu Zhang, Masood ur Rehman Kayani, Zhengting Wang, Yao Shen, Kenn Liu Su, Kouken Bielike, Lei Chen

**Affiliations:** ^1^ Center for Microbiota and Immunological Diseases, Shanghai Institute of Immunology, Shanghai Jiao Tong University School of Medicine, Shanghai, China; ^2^ Department of Gastroenterology, Ruijin Hospital, Shanghai Jiao Tong University School of Medicine, Shanghai, China

**Keywords:** 16S sequencing, wMGS sequencing, cholelithiasis, primary bile duct stone, secondary bile duct stone, biliary microbiota

## Abstract

**Introduction:**

Cholelithiasis has a high incidence worldwide and limited treatment options due to its poorly understood pathogenesis. Furthermore, the role of biliary microbiota in cholelithiasis remains understudied. To address these questions, we performed microbial sequencing from biliary samples from primary bile duct stone (PBDS) and secondary bile duct stone (SBDS) patients.

**Results:**

We analyzed in total 45 biliary samples, including those from cholelithiasis patients with PBDS or SBDS and people with other digestive diseases. 16S rRNA sequencing showed the bacteria family Alcaligenaceae increased in relative abundance in the lithiasis group compared with the non-lithiasis group. In addition, the PBDS group showed significantly lower bacterial diversity than SBDS, with Propionibacteriaceae, Sphingomonadaceae, and Lactobacillaceae as the most significant bacteria families decreased in relative abundance. We further performed whole metagenomic shotgun sequencing (wMGS) and found increased ability of biofilm synthesis and the ability to sense external stimuli in PBDS based on functional annotation of mapped reads. From genome-resolved analysis of the samples, we identified 36 high-quality draft bacterial genome sequences with completion ≥70% and contamination ≤10%. Most of these genomes were classified into Proteobacteria, Firmicutes, or Actinobacteria.

**Conclusions:**

Our findings indicated that there is a subtle impact on biliary microbiome from cholelithiasis while the difference is more pronounced between the PBDS and SBDS. It was revealed that the diversity of biliary microbiota in PBDS is lower, while some metabolic pathways are up-regulated, including those linked to higher incidence of different types of cancer, providing new insights for the understanding of cholelithiasis with different origin.

## Introduction

Cholelithiasis including gallbladder stone and bile duct stone has a prevalence rate of nearly 10% among adults (Tracy et al., 2021). In clinical practice, patients with bile duct stone can develop cholangitis due to bile duct obstruction and infection, which occurs frequently in emergencies (Williams et al., 2017). To date, endoscopic retrograde cholangiopancreatography (ERCP) remains the effective and minimally invasive treatment option for bile duct stone (Cianci and Restini, 2021). However, the relapse rate of bile duct stone is still high, up to 4-24% within 3-5 years, which can lead to patients’ suffering, increased medical expenditure, and risk of cholangiocarcinoma (Lujian et al., 2020).

Bile duct stone can be classified into primary bile duct stone (PBDS), in which case the stone predominately formed in the bile duct, or secondary bile duct stone (SBDS), which refers to the stone coming from the gallbladder. Differences exist in formation and composition of the two types of bile duct stone. SBDS is attributed to a combination of environmental and genetic causes, typically linked to cholesterol supersaturation. The primary constituent of SBDS is cholesterol, whereas it is calcium bilirubinate in the case of PBDS (Tazuma, 2006). However, the underlying pathogenesis of bile duct stones remains largely unknown, which makes it hard to prevent disease relapse (Reshetnyak, 2012).

With the rise of the next generation sequencing (NGS), the microbes living in the biliary tract have garnered increasing research attention. The biliary tract was initially considered sterile and microbe-free (Verdier et al., 2015), however, the application of NGS enabled the identification of distinct bacteria from the healthy biliary tract (Saltykova et al., 2016; Molinero et al., 2019). The presence of microorganisms in the bile suggests their possible influence on the normal function of the biliary system and even in pathogenesis of gallstones. According to a prevailing hypothesis, certain bacterial metabolites may interact with bile components to produce precipitation. Several previous studies have reported biliary microbiota in patients with cholelithiasis. Shen et al. (2015) studied the biliary microbiota of 15 cholelithiasis patients and identified common bacteria that usually exist in the oral cavity, and intestinal and respiratory tracts. By collecting bile from healthy liver donors, Molinero et al. (2019) used 16S sequencing to describe the differences in biliary microbiota between cholelithiasis patients and healthy people. The above studies confirmed the existence of biliary microbiota in healthy and diseased states and provided a preliminary description of the biliary microbiota. However, the microbiota differences between different types of bile stones such as PBDS and SBDS have never been revealed.

To address these questions, we conducted an initial analysis of a large cohort of cholelithiasis patients, along with non-lithiasis subjects with other digestive diseases requiring ERCP (pancreatitis, pancreatic tumors, etc.) to explore the microbial diversity and taxonomic differences using a combination of 16S rRNA and wMGS sequencing. In addition, lithiasis patients in this cohort were divided into PBDS and SBDS groups according to clinical diagnosis and their biliary microbiota compared. We further performed *de novo* assembly and carried out the genome-resolved analysis on the wMGS samples to recover a catalog of representative microbial genomes from the bile.

## Materials and Methods

### Patients and Bile Sample Collection

All patients were recruited at Shanghai Ruijin Hospital (affiliated with Shanghai Jiao Tong University School of Medicine, Shanghai). The diagnosis of cholelithiasis and the determination of primary or secondary bile duct stone were performed by trained clinicians from Shanghai Ruijin Hospital using CT, MRI, or ultrasound examinations. The indication for ERCP went beyond bile stones and included: (1) unexplained obstructive jaundice; (2) other biliary tract diseases such as tumors and sclerosing cholangitis; (3) congenital bile duct abnormality; and (4) pancreatic diseases such as tumor, chronic pancreatitis, etc. Therefore, the non-lithiasis group in this study consisted of patients who were determined to be free of cholelithiasis by the aforementioned diagnostic method and had other diseases. None of the patients had used any antibiotic at least two weeks prior to sample collection and no ongoing infections were detected at the procedure. A detailed patient description is provided in **Supplementary Materials** and **Supplementary Table S1**.

A bile sample (5-15mL) was extracted from the common bile duct during ERCP before application of contrast media, following the protocols presented in a previous study (Shen et al., 2015). Metronidazole was used to flush the endoscopic channel and operation area to avoid contamination before the bile sample was extracted using a sterile disposable cholangiography catheter, which was inserted through the endoscopy channel. Samples were immediately transported on ice and stored at -80°C until use. The protocol was reviewed by the Ethics Committee of Ruijin Hospital Affiliated with Shanghai Jiao University School of Medicine (2019-186).

### DNA Extraction and Sequencing

Metagenomic DNA was extracted from bile samples using PowerFecal DNA Isolation Kit (Qiagen), following manufacturer’s protocol. The quality of DNA was accessed using Agilent 2100 Bioanalyzer (Agilent Technologies, CA) and quantification was performed by Qubit (Thermo Fisher Scientific, Wilmington, DE) and DNA aliquots were prepared and stored in -20°C until use.

The V1-V3 variable region of 16S rRNA gene was amplified and sequenced on Illumina Miseq (Illumina, CA), using the 27F1 (AGAGTTTGATCCTGGCTCAG) and 534R (ATTACCGCGGCTGCTGG) primers, following the protocol used in the Human Microbiome Project. Paired-end reads with a length of 300 bp per read were generated.

For the wMGS library preparation, DNA extracted from the lithiasis group was used to construct the paired-end metagenomic libraries with the Nextera DNA Flex Library Prep kit (Illumina, CA). For the library preparation, the manufacturer’s protocol was used which included fragmentation and adapter ligation of the DNA, polymerization of the adapter-ligated library, and purification of the amplified library. Metagenomic sequencing of the purified library was performed using the Illumina Hiseq X Ten (Illumina, CA) sequencer, for generating paired-end reads with approximate length of 150 bp per read-end.

### 16S rRNA Sequence Analysis

The raw data from Miseq was analyzed using QIIME2 v2019.1 (Bolyen et al., 2019). Pre-processing steps, which included correction of PCR sequencing error, and removal of low-quality and chimera reads, were carried out using the default parameter of DADA2 procedure (as implemented in QIIME2). Representative sequences were aligned to the SILVA128 reference database, followed by classification to obtain the taxonomic information. Only samples with more than 5000 reads and non-singleton ASVs were used for downstream 16S rRNA analysis. When 16S data from previous studies were used in this study, their raw data, if available, were subject to the same analysis pipeline.

### wMGS Analysis

The wMGS analysis included conventional metagenomic analysis and genome-resolved metagenomic analysis, following the protocol in previously published studies (Nayfach et al., 2019; Vatanen et al., 2019). In brief, high quality and host removal reads were annotated using Kaiju (Menzel et al., 2016) with viral, fungal, and bacterial databases. MEGAHIT (Li et al., 2015) and FMAP (Kim et al., 2016) were used to assemble reads and annotate functional pathway abundance, respectively. After the assembly, the contigs were binned and refined by using the bin refinement module of MetaWRAP (Uritskiy et al., 2018). High quality bins were annotated as the metagenomically assembled genomes (MAGs) and used in downstream analysis. Protein and clusters of orthologous groups (COG) were obtained by using Prokka (Seemann, 2014) and eggNOG-mapper (Huerta-Cepas et al., 2017). For details on wMGS data analysis, see **Supplementary Methods**.

### Statistical Analysis

All statistical analyses were performed with R. Differences between groups were tested by Wilcoxon ranksum test, statistical significances were adjusted by Benjamini–Hochberg correction method when necessary, and FDR < 0.05 was considered to be significant.

The alpha and beta diversity analysis were calculated and presented using the phyloseq (McMurdie and Holmes, 2013) and microbiome package (http://microbiome.github.com/microbiome). Pielou’s Evenness, Observed OTU, and Shannon indexes were used to analyze the within sample diversity (alpha diversity). Principal coordinates analysis on the unweighted Unifrac distances and Bray Curtis dissimilarity were used to demonstrate differences between-sample diversity (beta diversity). PERMANOVA test in the vegan package (Oksanen et al., 2003) was used to investigate the association between microbiome composition and various clinical features.

The phylogenetic features that differed between all of cases were obtained by linear discriminant analysis effect size (LEfSe) (Segata et al., 2011) with an LDA score threshold of > 3 and the Kruskal-Wallis test (*α* < 0.05). ALDEx2 (Fernandes et al., 2013; Fernandes et al., 2014) aggregated ASVs according to the Order, Family, and Genus taxonomic hierarchy level with the Wilcoxon test (p < 0.05) used as a supplement to correct the possible deviation caused by small sample size.

## Results

### Biliary Microbiome in Cholelithiasis Shows Subtle but Defined Differences From the Control

**Figure 1:**
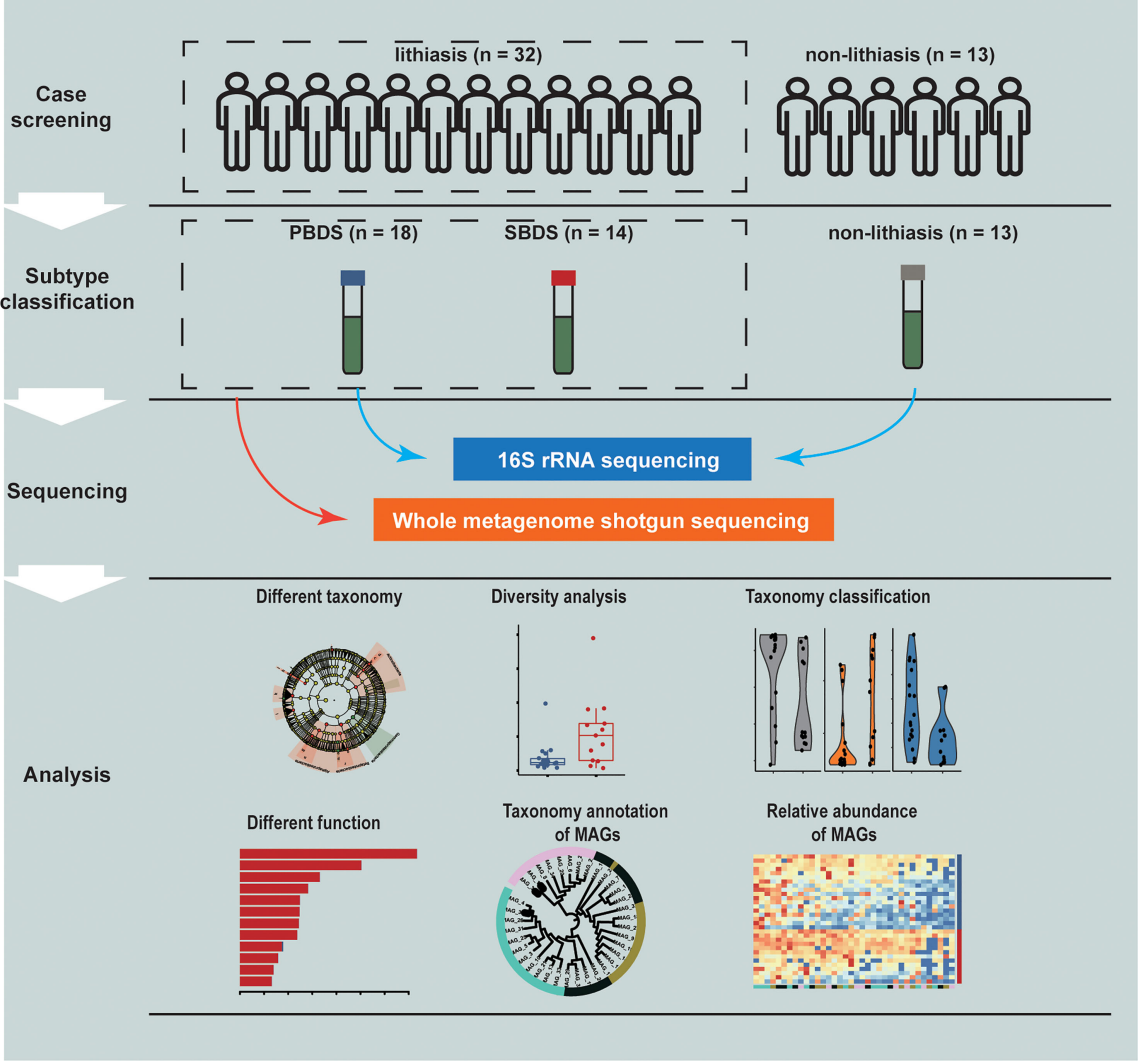
Study design. A total of 45 samples were collected and analyzed through 16S rRNA and wMGS sequencing to explore the differences of biliary microbiota in PBDS, SBDS, and non-lithiasis groups. wMGS, whole metagenomic shotgun sequencing; PBDS, primary bile duct stone; SBDS, secondary bile duct stone.

In total, 45 subjects were recruited at Ruijin Hospital in Shanghai, China, which can be divided into the lithiasis group (n=32) and the non-lithiasis group (n=13). Samples of the patients with or without lithiasis were all obtained after the completion of papillary intubation before cholangio-pancreatography and treatment. Furthermore, the lithiasis group can be divided into PBDS (n=18) and SBDS (n=14) according to clinical diagnosis. None of the subjects had prior history of gallbladder cancer (**Figure 1** and **Supplementary Table S1**).

We performed 16S rRNA sequencing, to determine the composition of the biliary microbiota, generating an average of 63,977 raw reads per sample (**Supplementary Table S2**). QIIME2 (Bolyen et al., 2019) was used to preprocess and classify the raw reads that generated a total of 5110 features which encompassed 21 different phyla with SILVA database. Proteobacteria, Firmicutes, Synergistetes, Bacteroidetes, Fusobacteria, and Actinobacteria were the most abundant bacterial phyla. The six were also the top six identified in a previous study by Shen et al. (2015). Taxonomic analysis at the order level indicated that Enterobacteriales were most abundant, whereas most of the other identified orders, including Lactobacillales, Fusobacteriales, and Bifidobacteriales (**Supplementary Table S3**), were also commonly found in the digestive tract. We then compared the alpha diversities (Pielou’s Evenness index) between the lithiasis and non-lithiasis group, which showed no significant difference (FDR-corrected *P* = 0.60) (**Supplementary Figure 1A**). Noting that our non-lithiasis subjects actually consisted of patients with digestive diseases other than cholelithiasis, we examined data from a recent study by Molinero et al. (2019), in which the control group contained healthy liver donors. We set the sample depth as 7920 reads/sample for rarefaction of both datasets. As expected, the healthy control group in Molinero et al. showed the highest alpha diversity, whereas their lithiasis group showed similar lower alpha diversity (Pielou’s Evenness index) with ours (FDR-corrected *P* = 0.61 and FDR-corrected *P* = 0.76, respectively) (**Supplementary Figure 1A**).

**Figure 2:**
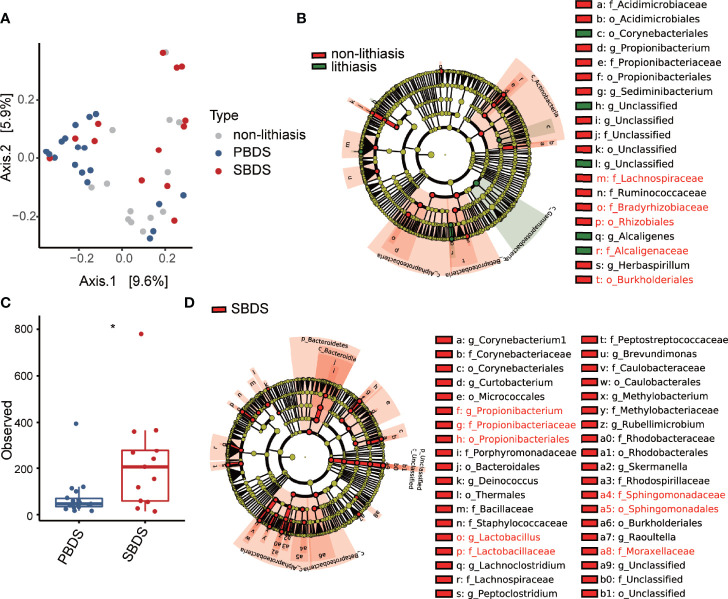
Diversity and differential abundance analysis by 16S sequencing. **(A) **Beta diversity analysis presented as principal-coordinate analysis (PCoA) o unweighted Unifrac distances. **(B)** The cladogram of taxonomic distribution of the lithiasis and non-lithiasis group, obtained by LEfSe with an LDA score threshold of > 3 (α < 0.05), those validated by ALDEx2 are marked red (Wilcoxon test; p < 0.05). **(C)** Alpha diversity in the form of Shannon index compared between PBDS and SBDS (Wilcoxon test; *, FDR < 0.05). **(D)** LEfSe analysis between SBDS and PBDS, parameters are the same as for panel **(B)**, those validated by ALDEx2 are marked red (Wilcoxon test; p < 0.05). PBDS, primary bile duct stone; SBDS, secondary bile duct stone.

We next examined the clinical variables contribution to microbiome variation using PERMANOVA using unweighted UniFrac distance as a measure of microbiome variation (**Table 1**). PERMANOVA analysis demonstrated that the distribution of biliary microbiota in the samples was independent of BMI, age, etc. Furthermore, the lithiasis status was not a significant factor either (PERMANOVA *R^2^* = 0.031; FDR-corrected *P* = 0.065). Meanwhile, the most significant contribution was attributed to the PBDS/SBDS classification (PERMANOVA *R^2^* = 0.069; FDR-corrected *P* = 0.013). In addition, the history of cholecystectomy also showed significant contribution (PERMANOVA R^2^ = 0.037; FDR-corrected *P* = 0.039). Using a combination of LEfSe (Segata et al., 2011) and ALDEx2 (Fernandes et al., 2013; Fernandes et al., 2014), several differentially abundant taxa between the lithiasis and the non-lithiasis group were identified. Bradyrhizobiaceae and Lachnospiraceae showed higher relative abundance in the non-lithiasis samples while Alcaligenaceae showed higher relative abundance in the lithiasis samples (**Figure 2B** and **Supplementary Figure 1C**), which is similar to the results of Molinero et al. *Alcaligenes recti* (family Alcaligenaceae) is known to be involved in the metabolism of various bile acids (Mazumder and Mahato, 1993), hence it is plausible that it plays an important role in the cholelithiasis patients.

**Table 1:** PERMANOVA based on unweighted UniFrac distance of different clinical information.

	Df	SumOfSqs	R2	F	Pr(>F)	FDR
**Cholelithiasis status**	1	0.614	0.031	1.356	0.020	0.065
**PBDS/SBDS/non-lithiasis**	2	1.361	0.069	1.528	0.001	0.013
**Prior cholecystectomy**	1	0.718	0.037	1.594	0.006	0.039
**Oncologic status**	1	0.624	0.032	1.379	0.014	0.061
**BMI**	1	0.519	0.026	1.141	0.140	0.303
**Prior ERCP/surgery or biliary intervention**	1	0.523	0.027	1.151	0.126	0.303
**Prior biliary infection**	1	0.510	0.026	1.120	0.176	0.327
**Pancreatic abnormality**	1	0.476	0.024	1.044	0.296	0.481
Comorbidities*	1	0.453	0.023	0.991	0.434	0.627
**Gender**	1	0.435	0.022	0.953	0.574	0.678
**Age**	1	0.440	0.022	0.964	0.528	0.678
**Antibiotic in last 3 months**	1	0.394	0.020	0.860	0.875	0.875
**Liver disease**	1	0.406	0.021	0.888	0.821	0.875

* The statistical analysis is based on the yes or no.

### The Biliary Microbiota of PBDS Has Lower Diversity and Different Functional Potential Compared With SBDS

Since our data indicated considerable distinction between PBDS and SBDS (**Figure 2A**), our following analysis focused on the comparison between the two groups. The alpha diversity (Observed OTU index) of the PBDS was significantly lower in contrast with the SBDS (FDR-corrected *P* = 0.015) (**Figure 2C**). A similar result was obtained using the Shannon index (FDR-corrected *P* = 0.062) (**Supplementary Figure 2A**). Next, we used the aforementioned approach to identify the differentially abundant bacteria between the two groups and found enrichment of several families in SBDS, including Propionibacteriaceae, Sphingomonadaceae, Lactobacillaceae, etc. (**Figure 2D** and **Supplementary Figure 2B**).

**Figure 3:**
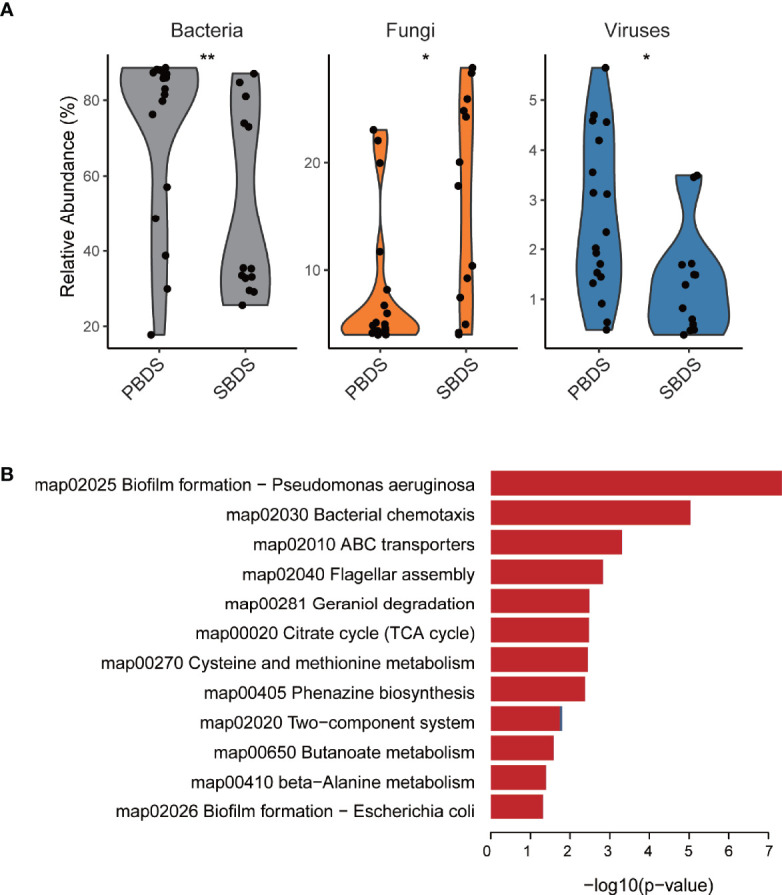
Taxonomic and functional analysis of the wMGS data. **(A)** The percentage of bacteria, fungi, and viruses in each metagenomic sample among SBDS and PBDS (Wilcoxon test; *, FDR < 0.05; **, FDR < 0.01). **(B)** Different pathways between SBDS and PBDS, red represents orthologs up-regulated in the PBDS, and blue represents down-regulated. wMGS, whole metagenomic shotgun sequencing; PBDS, primary bile duct stone; SBDS, secondary bile duct stone.

To further characterize the biliary microbiota of cholelithiasis patients, we performed metagenomic analysis on 31 of the lithiasis samples by wMGS sequencing, obtaining on average 46 million raw reads per sample. After quality control and removal of human DNA, metagenomic sequences were aligned to viral, fungal, and bacterial databases to obtain the taxonomic composition, and it was found that the relative abundance of each kingdom was different between the PBDS and SBDS (FDR-corrected *P* = 0.014, FDR-corrected *P* = 0.016, and FDR-corrected *P* = 0.0084, respectively) (**Figure 3A** and **Supplementary Table S4**). Among these reads, the proportion of viruses ranged from 0.2% to 5%, while the proportion of fungi ranged from 4.0% to 28.8%. The majority of the fungi were annotated as *Malassezia globosa*, a lipophilic and lipid-dependent yeast species, which may be detected on the skin surfaces (Gaitanis et al., 2012).

As expected, most of the reads were annotated as bacteria. We further performed *de novo* metagenomic co-assembly, obtaining 152,226 contigs with a total length of 361Mbp. Through functional annotation we identified a total of 4926 KEGG orthologs from the assembly. With ortholog comparison between the two groups, we found that not only the diversity decreased, but also several functional pathways were relatively downregulated in the PBDS. Biofilm formation and two-component system were the top two downregulated pathways. Other downregulated pathways included ones associated with transporters and compound metabolism (**Figure 3B** and **Supplementary Table S5**).

### Reconstructed Genomes From Human Biliary Metagenomes Pinpoint the Taxons Enriched in SBDS

**Figure 4:**
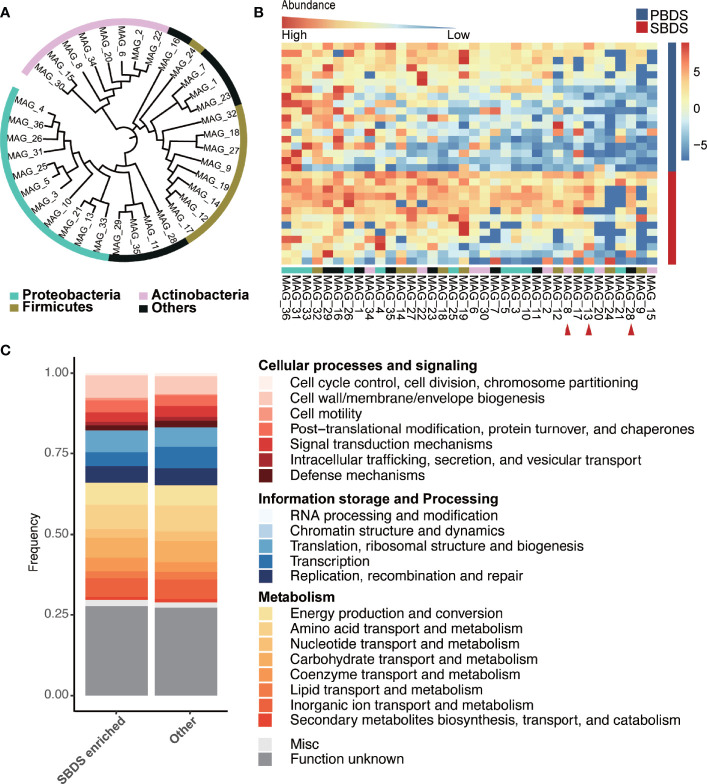
Taxonomic and functional analysis of the MAGs. **(A)** The taxonomic tree of the MAGs, annotated at the phylum level. The tree was generated using PhyloPhlAn and visualized by interactive tree of life (iTOL) webserver (https://itol.embl.de/). **(B)** Relative abundance of the MAGs in the SBDS and PBDS samples, ordered by their average relative abundances, the red arrow points to the MAGs with significantly higher relative abundance in SBDS (Wilcoxon test; p < 0.05). **(C)** COG profiles of the SBDS enriched MAGs and other MAGs. MAGs: metagenomically assembled genomes; COG, clusters of orthologous group; PBDS, primary bile duct stone; SBDS, secondary bile duct stone.

To recover individual microbial genomes from human bile, we performed subsequent genome binning and refinement using the *de novo* assembly. A total of 36 metagenomically assembled genomes (MAGs) with variable genome quality were obtained. Among these, 20 MAGs showed completion above 90% (**Supplementary Table S6**). The genomes had a total length that ranged from ~1.6 Mbp (MAG_23) to ~7.3 Mbp (MAG_10) and showed N50 in the range of ~3 Kbp (MAG_33) to 716 Kbp (MAG_25), with the genome sizes of most MAGs in the range between 1 and 4 Mbp (**Supplementary Figure 3A** and **Supplementary Table S7**). The minimum and maximum GC% of the MAGs were 26.65% and 72.78%, respectively (**Supplementary Figure 3B**). All MAGs were classified as Bacteria, 11 MAGs were assigned to the phylum Proteobacteria, 9 to Firmicutes, and 8 to Actinobacteria, these three phyla also showed higher relative abundance in the 16S rRNA sequencing data, other recovered phyla included Fusobacteria (3 MAGs), Bacteroidetes (3 MAGs), Synergistetes (1 MAG), and Verrucomicrobia (1 MAG) (**Figure 4A**). A total of 15 genera were detected, including: *Clostridium, Fusobacterium, Actinomyces, Haemophilus, Propionibacterium, Streptococcus, Aeromonas, Campylobacter, Methylobacterium, Eggerthella, Bifidobacterium, Prevotella, Klebsiella, Bacteroides*, and *Enterococcus*, all of them have been reported in the digestive tract (Rajilic-Stojanovic and de Vos, 2014; Kose et al., 2018). Among the detected genera, *Propionibacterium* is considered as a biomarker for the non-mucosal site of the digestive tract (Segata et al., 2012). This finding validates our data and approach again. Eleven of the 36 MAGs were successfully identified at the species level, i.e., MAG_8 classified as *Propionibacterium acnes* (**Supplementary Table S8**). Among the identified species, *B. animalis* (MAG_34) is a common probiotic, while *F. nucleatum* (MAG_23) has been reported to be a pathogen (Struve and Krogfelt, 2004; Lahti et al., 2014; Hidalgo-Cantabrana et al., 2017; Yu et al., 2017). Moreover, *H. parainfluenzae* (MAG_4) is a marker of the upper respiratory tract microbiota and is reported to be related to biliary tract infection (Frankard et al., 2004), demonstrating the complexity of the biliary environment in cholelithiasis patients.

We compared the relative abundance of MAGs between SBDS and PBDS. The MAGs were arranged according to their prevalence in the PBDS, and it showed that the MAGs differing in relative abundance between the two groups were mainly concentrated on the right side, which means that these MAGs were the ones that existed in only a few PBDS samples (**Figure 4B** and **Supplementary Table S9**). The three differing MAGs, named MAG_28, MAG_13, and MAG_8, were enriched in SBDS, and classified as Verrucomicrobiaceae, *Campylobacter*, and *Propionibacterium acnes*, respectively. This result agreed with the lower microbiota diversity in PBDS cases.

### Functional Annotation of the Human Biliary Genomes

Approximately 1440-7126 coding sequences (CDS) were predicted in the bile MAGs, the lowest number of CDS was detected in MAG_23 and the largest was in MAG_10 (**Supplementary Table S10**). The number of transfer RNAs (tRNA) predicted in each MAG ranged from 12 to 61, the number of ribosomal RNAs (rRNA) ranged from 0 to 7, and the transferred messenger RNAs (tmRNA) were no more than one. The total number of gene families in all MAGs was 7385, and the most abundantly identified family was ABC transporter ATP-binding protein, which is essential in cell viability, followed by fumarate reductase flavoprotein subunit, which is involved in anaerobic respiration mainly found in *Escherichia coli*, and 3-oxoacyl-[acyl-carrier-protein] reductase FabG, which is involved in the fatty acid biosynthetic process. We obtained 2435 different clusters of orthologous groups (COGs) among the predicated coding sequences of the 36 MAGs. COG0745 was most abundant in all MAGs, followed by COG1609 and COG2197, which are annotated as the DNA-binding response regulator, DNA-binding transcriptional regulator, and DNA-binding response regulator. The majority of the known functions were categorized to amino acid transport and metabolism (COG code E), followed by transcription (K); carbohydrate transport and metabolism (G); energy production and conversion (C); and inorganic ion transport and metabolism (P). MAG_15 showed a strong metabolic capacity because it had higher relative abundance of energy production and conversion (C) pathways. MAG_12 and MAG_17, which could only be classified as Enterococcaceae, contained higher relative abundances of carbohydrate transport and metabolism (G) pathways (**Supplementary Figure 3C**). We found that the three SBDS enriched MAGs to have higher rates of cellular processes and signaling, while other MAGs have higher rates of information storage and processing, especially coenzyme transport and metabolism (Wilcoxon test, p = 0.028; **Figure 4C**).

## Discussion

In this study, we provided a large biliary microbiome cohort complete with both 16S sequencing and wMGS data and investigated the microbiome heterogeneity behind different types of cholelithiasis. We confirmed the bacteria phyla of Fusobacteria, Bacteroidetes, Synergistetes, Firmicutes, Proteobacteria, and Actinobacteria as the most abundant phyla in bile, in accordance with results previously obtained by Shen et al. (2015) and Molinero et al. (2019). By comparing the biliary microbiota of the lithiasis and non-lithiasis group, we observed that Alcaligenaceae reached higher relative abundance among the lithiasis samples. Within this family, *Alcaligenes recti* has been reported to be related to the metabolism of various bile acids (Mazumder and Mahato, 1993).

Within the lithiasis group, the alpha diversity of PBDS was significantly lower than that of the SBDS. At the same time, we found Propionibacteriaceae, Sphingomonadaceae, and Lactobacillaceae were highly enriched in the SBDS. The presence of these bacteria makes the biliary tract environment in SBDS more similar to the normal digestive tract. Lactobacillaceae is one of the most common probiotics (Gorbach, 1990), and has also been reported to be involved in the prevention of colon cancer (Meehan and Beiko, 2014; Torres-Maravilla et al., 2021). While interestingly, *Peptoclostridium difficile*, which was first isolated from the stool of healthy infants, is a nosocomial pathogen worldwide causing antibiotic-associated diarrhea and also tends to have higher relative abundance in SBDS (Brook, 2008) (**Supplementary Figure 2C**). Overall, the biliary microbiota of SBDS patients showed a more complex and closer to normal bacterial composition, an interesting difference from PBDS. Using PERMANOVA, various patient factors were examined in terms of their contribution to biliary microbiome variation and PBDS/SBDS dichotomy was found to be the most significant factor. Cholecystectomy history came in as second. However, caution should be taken as cholecystectomy was highly correlated with PBDS in our cohort.

Only a few previous studies ever described the function of biliary microbiota. Shen et al. (2015) compared the function of cholelithiasis patients’ biliary microbiota with fecal samples from the Human Microbiome Project and found that flagellum assembly function was significantly up-regulated. These biliary microbiota specific pathways also showed significant difference between PBDS and SBDS (**Supplementary Table S5**). It has been reported that the formation of bacterial biofilm is significantly associated with the occurrence and development of various cancers (Rizzato et al., 2019). The low diversity and complex metabolic environment of PBDS suggest a stronger association with other malignancies.

However, this study was not without limitations. As ERCP is an invasive procedure and it would be impossible to get healthy bile this way, the non-lithiasis group in our study was actually the patients with other digestive diseases requiring this procedure. To compensate for this, we compared biliary microbiota data with healthy controls from a previous study (Molinero et al., 2019). On the other hand, as patients with PBDS or SBDS in this study have complex backgrounds and may have different previous medical histories, such as cholecystectomy, the conclusions should be treated with more caution. That is, although the differences between the PBDS and SBDS are significant, the influence of other factors cannot be ruled out. In addition, although we excluded patients with antibiotics use within two weeks before ERCP, some of them did receive an unknown type of antibiotic within three months, which could cause some bias in the results. Nevertheless, PERMANOVA analysis suggested this particular effect to be relatively small (PERMANOVA R^2^ = 0.020; FDR-corrected *P* = 0.875). Finally, although the sample size of our study was relatively small compared to general fecal microbiome studies, which may result in only effects with larger size to show significance, we did get some important differences between different groups, nonetheless. Limited by the sample size, there may be some signals that have not been confirmed, which can benefit from future studies with additional samples. Still, we would argue that as the biliary microbiome is generally less diverse and more stable, as can be seen from study accordance, compared to the fecal microbiome, similar statistical power would be reached with less samples. Also, to suppress spurious signals from a small sample size, we used two different differential abundance analysis tools, LEfSe and ALDeX2 to identify biomarkers. Overall, the information gained from bioinformatics still needs to be viewed with caution and validated by further functional experiments.

Collectively, the taxonomic annotation and microbiome diversity suggest that biliary microbiota is more diverse in SBDS than in PBDS, and functional annotation shows that multiple metabolic pathways are up-regulated in PBDS, showing the possible relation between PBDS and different types of other malignant diseases. Our study, which was the first to perform *de novo* assembly and carried out the genome-resolved analysis of the biliary microbiota to explore PBDS and SBDS, would help us to understand these two cholelithiasis types and pave the way for exploring the microbiota-cholelithiasis connection in the future.

## Data Availability Statement

The datasets presented in this study can be found in online repositories. The names of the repository/repositories and accession number(s) can be found below: https://www.ncbi.nlm.nih.gov/, PRJNA580086.

## Ethics Statement

The studies involving human participants were reviewed and approved by Ethics Committee of Ruijin Hospital Affiliated to Shanghai Jiaotong University School of Medicine (2019-186). The patients/participants provided their written informed consent to participate in this study.

## Author Contributions

RF performed the experiments, data analysis, and prepared the manuscript. Bile samples were collected by KB and TZ. MK provided support in the WMGS analysis and manuscript revision. LC and ZW supervised the study and edited the manuscript. All authors have read and approved the manuscript.

## Funding

This investigation was supported by National Natural Science Foundation of China (No. 81670503 and 81802906), and Shanghai Jiao Tong University, School of Medicine New PI Startup Fund (No. 17X100040046), and Shanghai Sailing Program (No. 20YF1428200).

## Conflict of Interest

The authors declare that the research was conducted in the absence of any commercial or financial relationships that could be construed as a potential conflict of interest.

## Publisher’s Note

All claims expressed in this article are solely those of the authors and do not necessarily represent those of their affiliated organizations, or those of the publisher, the editors and the reviewers. Any product that may be evaluated in this article, or claim that may be made by its manufacturer, is not guaranteed or endorsed by the publisher.
