# Supplementary material for: Patients with Primary and Secondary Bile Duct Stones Harbor Distinct Biliary Microbial Composition and Metabolic Potential
Source: Front Cell Infect Microbiol. 2022 Apr 25;12:881489. doi: 10.3389/fcimb.2022.881489 (PMC9082501; doi:10.3389/fcimb.2022.881489)
Supplement: Supplementary file 1 [file Table_1.docx]

Supplementary Material

## Supplementary Methods

**Diagnostic criteria**

The diagnosis of lithiasis was confirmed by trained clinician from Shanghai Ruijin Hospital using CT, MRI or ultrasound. The assignment of primary or secondary bile duct stone was according to the following criteria: 1) If the patient had history of cholecystectomy, the stone in bile duct should be PBDS; 2) If the patient had gallbladder, it would then be determined by radiological findings: 2a) If the patient did not have gallbladder stone, it would be PBDS; 2b) If the patient had gallbladder stone, radiological examinations would be used by an experienced clinician to compare the number, size and other morphological features of the stone in bile duct and gallbladder, combined with pathological status of the bile duct system, eg: diverticulum, stenosis, to determine if the stone in the bile duct was derived from the one in the gallbladder, or not.

**wMGS analysis**

1. **Shotgun metagenomic analysis**

Raw metagenomic reads with low quality (Q<20) were filtered by sickle (<https://github.com/najoshi/sickle>), and adapter sequences and ambiguous bases (N) were also trimmed by Trim Galore! (<https://github.com/FelixKrueger/TrimGalore>). Then, clean reads were aligned to the Human genome (hg38) using KneadData (<http://huttenhower.sph.harvard.edu/kneaddata>) and all the mapped reads were removed. Kaiju (Menzel et al., 2016) was used to annotate the taxonomy of the clean reads with viral, fungal and bacterial databases, which were downloaded from Kaiju website at 2019.10. As for the *de novo* assembly, MEGAHIT assembler (Li et al., 2015) was used to co-assemble all the reads with k-mers ranging from 27 to 147 and minimum contig length of 500 bp. Functional pathway abundance and difference was calculated by FMAP (Kim et al., 2016) with UniRef50 reference database.

1. **Genome-resolved metagenomic analysis**

After the assembly, the contigs were binned by MetaBAT2 (Kang et al., 2019) as well as MaxBin2 (Wu et al., 2016) with default parameters, and were refined by using the bin refinement module of MetaWRAP (Uritskiy et al., 2018). High quality bins were annotated as the metagenomically-assembled genomes (MAGs), which were assessed by CheckM v0.9.7 (Parks et al., 2015) with completion level ≥ 70% and contamination ≤ 10%, and used in downstream analysis. Quality and features of the MAGs (i.e. total length, N50, and number of contigs, and GC%) were visualized using QUAST (Gurevich et al., 2013) for the genome assemblies. Protein and clusters of orthologous groups (COG) were obtained by using Prokka (Seemann, 2014) and eggNOG-mapper (Huerta-Cepas et al., 2017).

Gurevich, A., Saveliev, V., Vyahhi, N., and Tesler, G. (2013). QUAST: quality assessment tool for genome assemblies. *Bioinformatics* 29(8)**,** 1072-1075. doi: 10.1093/bioinformatics/btt086.

Huerta-Cepas, J., Forslund, K., Coelho, L.P., Szklarczyk, D., Jensen, L.J., von Mering, C., et al. (2017). Fast Genome-Wide Functional Annotation through Orthology Assignment by eggNOG-Mapper. *Mol Biol Evol* 34(8)**,** 2115-2122. doi: 10.1093/molbev/msx148 %/ (c) The Author 2017. Published by Oxford University Press on behalf of the Society for Molecular Biology and Evolution.

Kang, D.D., Li, F., Kirton, E., Thomas, A., Egan, R., An, H., et al. (2019). MetaBAT 2: an adaptive binning algorithm for robust and efficient genome reconstruction from metagenome assemblies. *PeerJ* 7**,** e7359. doi: 10.7717/peerj.7359.

Kim, J., Kim, M.S., Koh, A.Y., Xie, Y., and Zhan, X. (2016). FMAP: Functional Mapping and Analysis Pipeline for metagenomics and metatranscriptomics studies. *BMC Bioinformatics* 17(1)**,** 420. doi: 10.1186/s12859-016-1278-0.

Li, D., Liu, C.M., Luo, R., Sadakane, K., and Lam, T.W. (2015). MEGAHIT: an ultra-fast single-node solution for large and complex metagenomics assembly via succinct de Bruijn graph. *Bioinformatics* 31(10)**,** 1674-1676. doi: 10.1093/bioinformatics/btv033 %/ (c) The Author 2015. Published by Oxford University Press. All rights reserved. For Permissions, please e-mail: journals.permissions@oup.com.

Menzel, P., Ng, K.L., and Krogh, A. (2016). Fast and sensitive taxonomic classification for metagenomics with Kaiju. *Nat Commun* 7**,** 11257. doi: 10.1038/ncomms11257.

Parks, D.H., Imelfort, M., Skennerton, C.T., Hugenholtz, P., and Tyson, G.W. (2015). CheckM: assessing the quality of microbial genomes recovered from isolates, single cells, and metagenomes. *Genome Res* 25(7)**,** 1043-1055. doi: 10.1101/gr.186072.114 %/ (c) 2015 Parks et al.; Published by Cold Spring Harbor Laboratory Press.

Seemann, T. (2014). Prokka: rapid prokaryotic genome annotation. *Bioinformatics* 30(14)**,** 2068-2069. doi: 10.1093/bioinformatics/btu153.

Uritskiy, G.V., DiRuggiero, J., and Taylor, J. (2018). MetaWRAP-a flexible pipeline for genome-resolved metagenomic data analysis. *Microbiome* 6(1)**,** 158. doi: 10.1186/s40168-018-0541-1.

Wu, Y.W., Simmons, B.A., and Singer, S.W. (2016). MaxBin 2.0: an automated binning algorithm to recover genomes from multiple metagenomic datasets. *Bioinformatics* 32(4)**,** 605-607. doi: 10.1093/bioinformatics/btv638 %/ (c) The Author 2015. Published by Oxford University Press. All rights reserved. For Permissions, please e-mail: journals.permissions@oup.com.

## Supplementary Figures


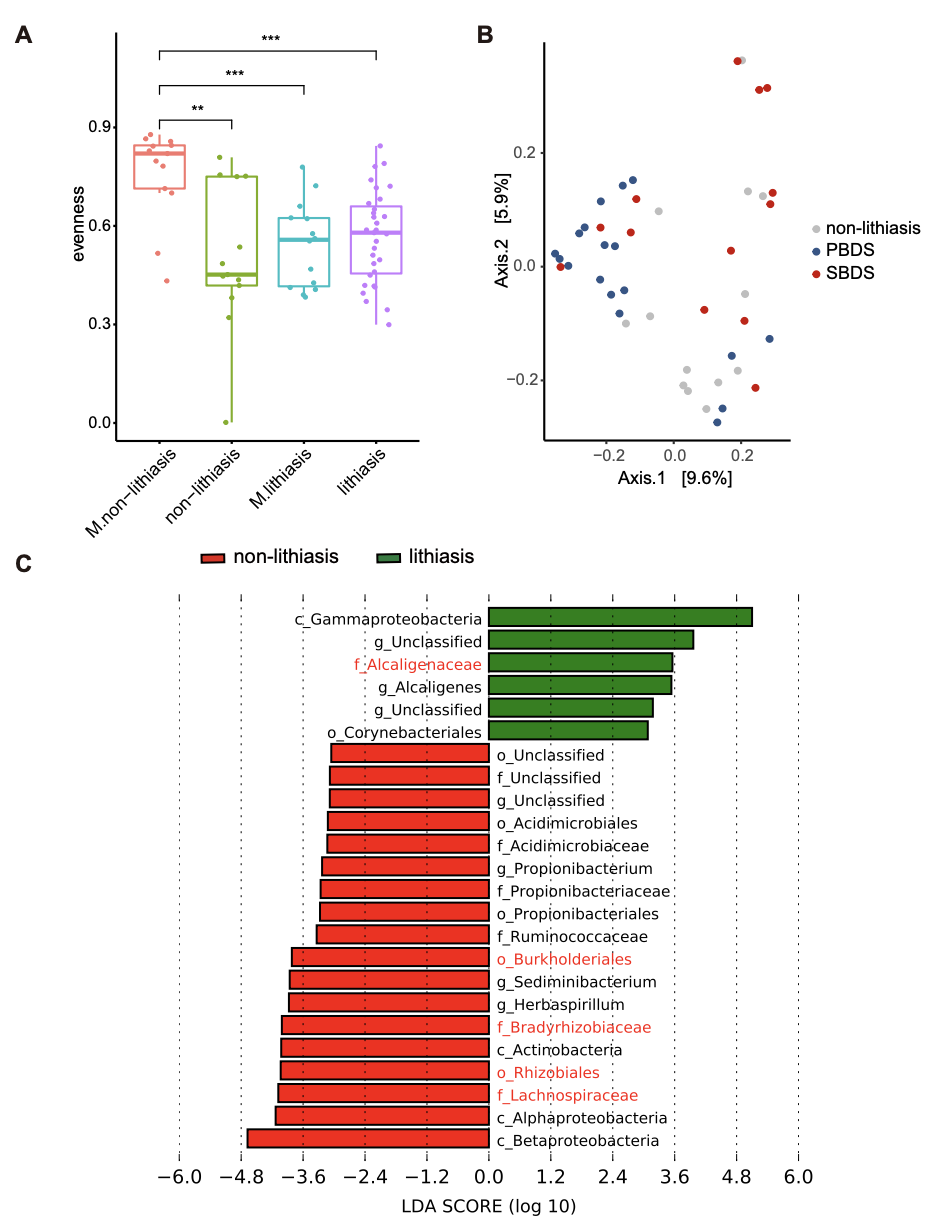


**Supplementary Figure 1:** **Diversity and differential abundance analysis by 16S sequencing among all samples.** **(A)** Pielou’s Evenness index values compared with data from Molinero et al. (Wilcoxon test; *, FDR < 0.05; **, FDR ≤ 0.01; ***, FDR ≤0.001). The four groups from left to right are: 1) non-lithiasis group from Molinero et al.; 2) non-lithiasis group in this study; 3) lithiasis group from Molinero et al.; 4) lithiasis group in this study. **(B)** Beta diversity analysis presented as principal-coordinate analysis (PCoA) with Bray-Curtis dissimilarity. **(C)**The [LEfSe](http://huttenhower.sph.harvard.edu/galaxy/tool_runner?tool_id=testtoolshed.g2.bx.psu.edu%2Frepos%2Fgeorge-weingart%2Flefse%2FLEfSe_cla%2F1.0) results of taxonomic distribution of lithiasis and non-lithiasis group, obtained by LEfSe with an LDA score threshold of > 3 (α < 0.05), those validated by ALDEx2 marked red (Wilcoxon test; p < 0.05).


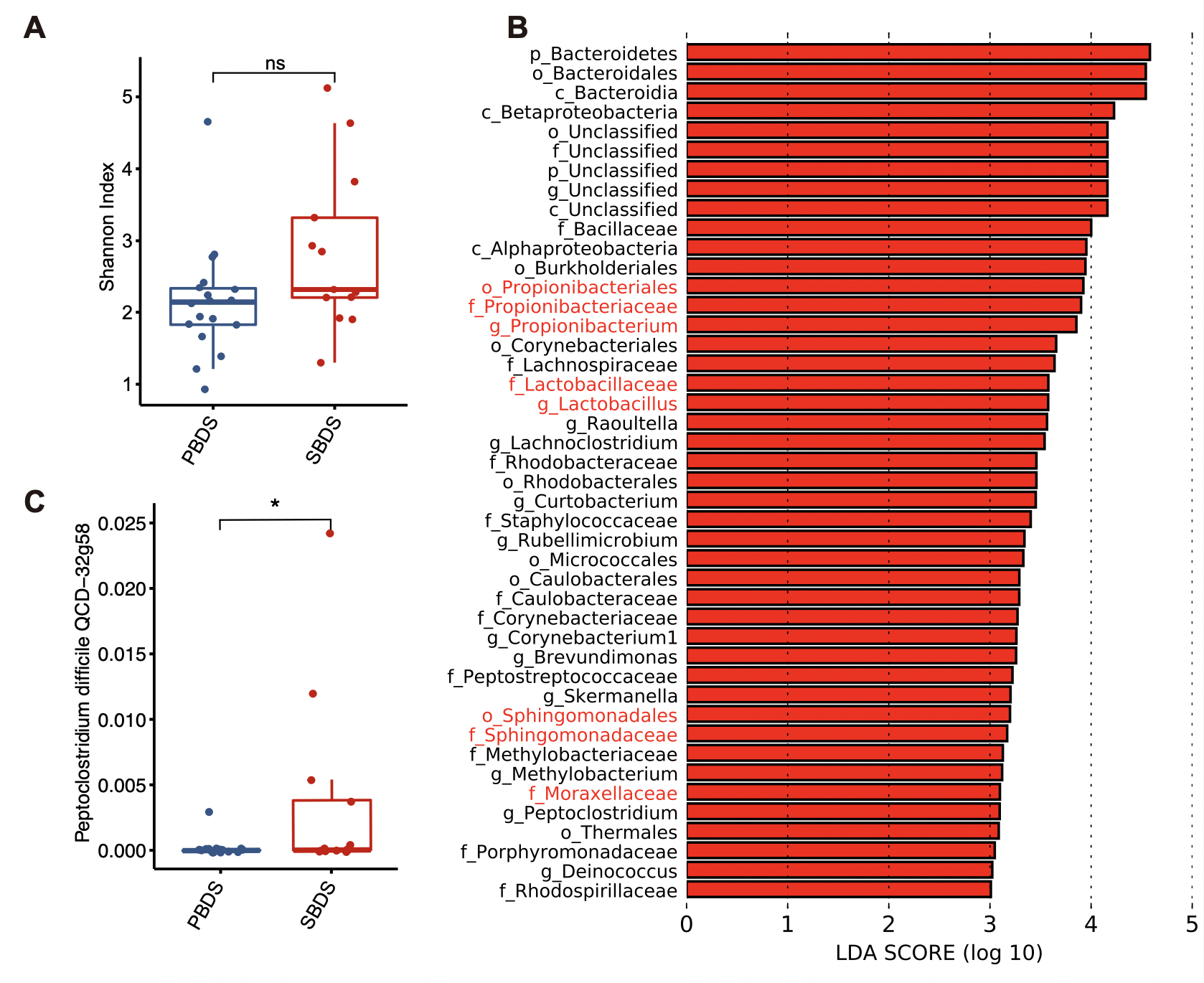


Supplementary Figure 2: Diversity and differential abundance analysis by 16S sequencing between PBDS and SBDS. (A) Observed OTUs alpha diversity index values between PBDS and SBDS (Wilcoxon test; ns, not significant). (B) The [LEfSe](http://huttenhower.sph.harvard.edu/galaxy/tool_runner?tool_id=testtoolshed.g2.bx.psu.edu%2Frepos%2Fgeorge-weingart%2Flefse%2FLEfSe_cla%2F1.0) results of taxonomic distribution of SBDS and PBDS, obtained by LEfSe with an LDA score threshold of > 3 (α < 0.05), those validated by ALDEx2 marked red (Wilcoxon test; p < 0.05). (C) Relative abundance of *Peptoclostridium difficile QCD−32g58* in PBDS and SBDS (Wilcoxon test; *, FDR < 0.05).


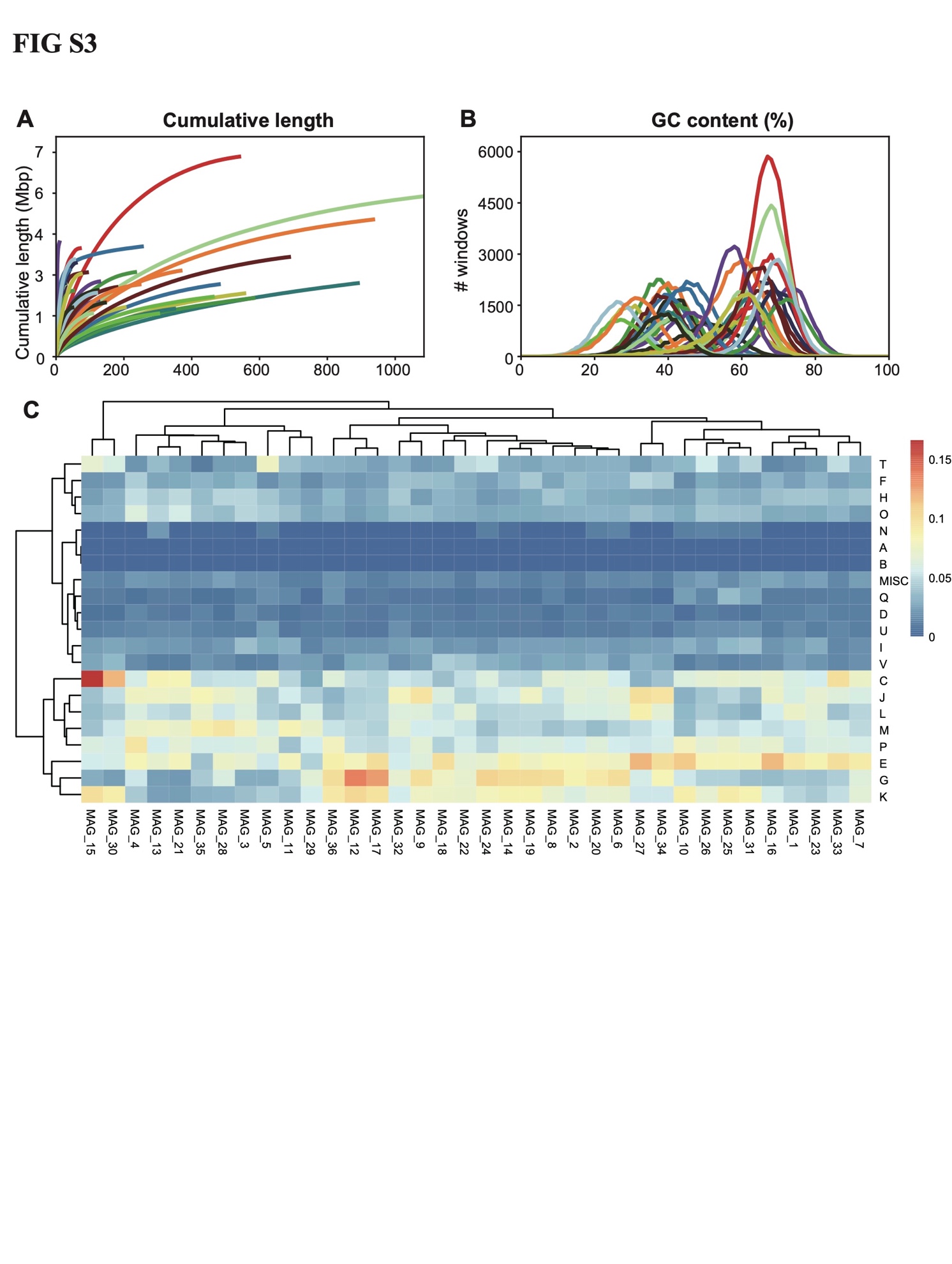


**Supplementary Figure 3:** **Characterization of the MAGs. (A)** Cumulative length of the MAGs (y-axis) against the contig index (x-axis). **(B)** Distribution of the %GC content. **(C)**Relative abundance of the COG categories in each MAG.
